# Nickel and Cobalt Recovery from Spent Lithium-Ion Batteries via Electrodialysis Metathesis

**DOI:** 10.3390/membranes15040097

**Published:** 2025-03-25

**Authors:** Adam Isaksson, Juan Anaya Garzon, Ida Strandkvist, Lena Sundqvist Öqvist

**Affiliations:** 1 Division of Minerals and Metallurgical Engineering, Luleå University of Technology, 971 87 Luleå, Sweden; ida.strandkvist@ltu.se (I.S.); lena.sundqvist-oqvist@ltu.se (L.S.Ö.); 2 Northvolt Revolt AB, 721 36 Västerås, Sweden; juandavid.anaya-garzon@northvolt.com

**Keywords:** lithium-ion batteries, recycling, black mass, EDTA, electrodialysis

## Abstract

Recycling of spent lithium-ion batteries is important due to the increasing demand for electric vehicles and efforts to realize a circular economy. There is a need to develop environmentally friendly processes for the refining of nickel, cobalt, and other metals contained in the batteries. Electrodialysis is an appealing method for recycling of battery metals with selective separation and low chemical input. In this study, sodium sulfate was used in an electrodialysis metathesis procedure to sequentially separate EDTA-chelated nickel and cobalt. Replacing hitherto used sulfuric acid with sodium sulfate mitigates membrane fouling caused by precipitation of EDTA. It was possible to separate up to 97.9% of nickel and 96.6% of cobalt at 0.10 M, a 30-times higher concentration than previously reported for electrodialysis of similar solutions. Through the thermally activated persulfate method, new to this application, 99.7% of nickel and 87.0% of cobalt could be precipitated from their EDTA chelates. Impurity behavior during electrodialysis of battery leachates has not previously been described in the literature. It is paramount to remove copper, iron, and phosphorous prior to electrodialysis since they contaminate the nickel product. Aluminum was difficult to remove in the solution purification step and ended up in all electrodialysis products.

## 1. Introduction

The green transition of society has led to a greater demand for batteries since they are essential for the production of electric vehicles (EVs) and energy storage solutions. The lithium-ion battery (LIB) is the most common type of battery in rechargeable products owing to its high energy density, great long-term performance, and reliability [1]. One main LIB chemistry employs NMC (Nickel Manganese Cobalt) as cathode active material. The world market demand for lithium (Li), nickel (Ni), and cobalt (Co) for EV is estimated to exceed the total raw material production of these metals in 2040. It has even been suggested that the need for Li and Co might reach eight times the production volumes in that same year [2]. For this reason, it is paramount to optimize and develop sustainable recycling processes.

In hydrometallurgical recycling, LIBs are first discharged, milled, sieved, and treated by other mechanical methods to separate the plastic casing and electrode materials made of copper (Cu) and aluminum (Al). What remains is a fine black powder commonly referred to as black mass. It contains considerable amounts of graphite and most of the valuable metals—Li, Ni, Mn, Co—and is commonly leached with a mixture of 2–4 M sulfuric acid (H_2_SO_4_) and 1–5% (*v*/*v*) hydrogen peroxide (H_2_O_2_) at 60–80 °C. After removal of the solids by filtration, the leachate is commonly refined by solvent extraction (SX) [3,4,5,6]. This method enables large throughputs at high selectivity but is relatively expensive, chemical-intense, and uses fossil, flammable solvents [7], contributing significantly to the overall environmental impact of the recycling process [8]. A possible alternative to SX for refining of LIB leachates is electrodialysis, which has a lower risk of a negative impact on the environment and occupational safety [9]. Electrodialysis utilizes an electric field across an arrangement of cation-exchange membranes (CEM) and anion-exchange membranes (AEM) between two electrodes to transfer ions between different aqueous solutions [10,11].

Recent studies on electrodialysis for LIB recycling mostly deal with Li extraction from NMC leachates [12,13,14,15,16,17,18,19,20]. Fewer studies concern selective separation of Ni, Co, and Mn by electrodialysis. Chaudhary et al. [21] added ethylenediaminetetraacetic acid (EDTA) to remove Ni from a cobalt sulfate (CoSO_4_) solution by electrodialysis, and their results were later validated by Tzanetakis et al. [22]. This was possible due to the greater stability of the EDTA chelates with Ni^2+^ compared to Co^2+^. They reported an almost complete removal of Ni with approximately 20% molar excess of EDTA. There was a tradeoff between Ni selectivity and recovery depending on the EDTA addition, as more EDTA gave a higher Ni recovery but also co-recovery of Co. The separation of Ni and Co was also studied by Labbe et al. [23]. They used EDTA for selective removal of Ni followed by dechelation at a low pH due to the lower solubility of EDTA chelates in acidic media. It was possible to recover 84.5% of Ni from the chelate after 10 days by equivolumetric addition of nitric acid (HNO_3_) at −30 °C. Chan et al. [13,24,25] studied the separation of Ni, Co, Mn, and Li in a three-stage electrodialysis process with both a synthetic leachate and different black mass leachates. They added EDTA at 10% molar excess at pH 2 to selectively recover Ni at a concentration of 3.33 mM. EDTA at 20% molar excess was then added at pH 3 to recover Co, also at 3.33 mM. They reported a near-complete separation of Ni (>99%) in the Ni recovery stage and about 87% separation of Co in the Co recovery stage. Dechelation was performed by adding H_2_SO_4_ to precipitate EDTA at pH < 0.5. Like other studies [21,23], some Co reported to the Ni product when the Ni recovery was maximized. Similarly, Mn reported to the Co product. These elements were removed by selective precipitation. Taghdirian et al. [26] compared electrodialysis with electrodeionization for Ni/Co separation, and Siekierka et al. [27] studied ion-selective membranes for selective Co^2+^ extraction.

In previous studies, separation generally took place at millimolar concentrations to prevent reactions causing precipitation and subsequent membrane fouling. When the concentrations of divalent ions are high, there is a risk of metal hydroxide or sulfate precipitation. The concentration gradient formed in the diffusive layer close to the CEM surface increases the risk of precipitation and fouling [10,11]. This effect has caused problems in previous studies, such as for the separation of rare earth elements (REEs) [28]. Another type of precipitation is that of the chelating agent, since EDTA and many other agents show lower solubility at an acidic pH. The low solubility of EDTA has been shown experimentally; for example, by Pinto et al. [29].

**Figure 1:**
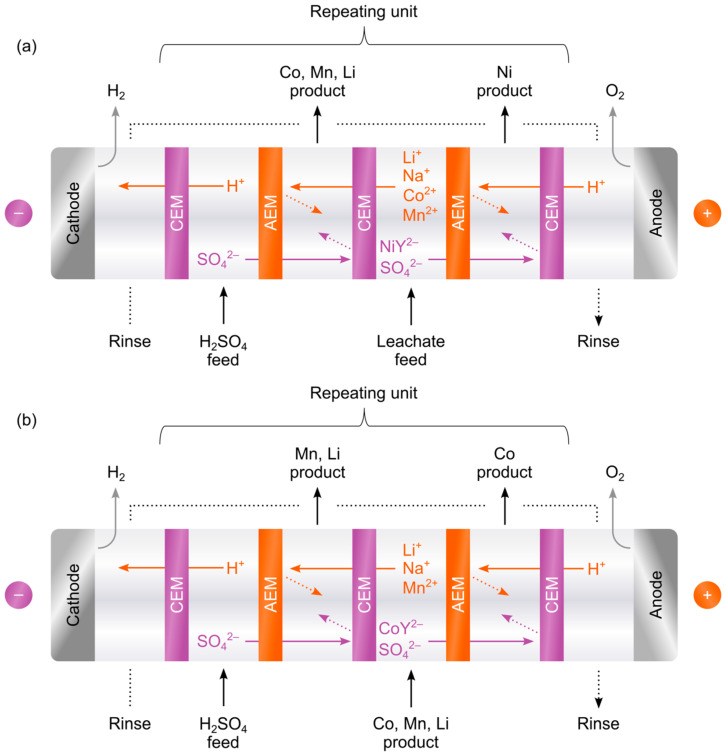
Examples of (**a**) Ni separation and (**b**) subsequent Co separation using a chelating agent (Y^4−^) in a metathesis process with a H_2_SO_4_ solution, cation exchange membranes (CEM) and anion exchange membranes (AEM), as reported in the literature. Transfer of H^+^ into the Ni and Co products causes a decrease in pH, precipitating Y^4−^ as H_4_Y (EDTA solids).

Electrodialysis for metal separation employs the exchange of ions between two feeds, forming two products, as seen in Figure 1. This type of process is called electrodialysis metathesis, meaning cations and anions in two salt solutions are recombined, forming two new salt solutions. As the leachate-chelating agent mixture constitutes the primary feed, a secondary feed is required for the recombination of ions. Previous studies primarily used H_2_SO_4_ for this purpose. As can be seen in Figure 1, hydrogen ions (H^+^, alternatively, hydronium ions H_3_O^+^) migrate from the H_2_SO_4_ feed to meet negatively charged Ni or Co chelates and sulfate ions, ensuring electrical neutrality. The pH rapidly decreases in the chelate product chamber, eventually causing precipitation of EDTA, thus limiting product concentration. One way to prevent this effect is to use an alkali such as sodium hydroxide (NaOH) as the starting electrolyte in the Ni recovery chamber, though this is problematic since ion exchange membranes are generally less stable in alkaline media [30,31]. Alternatively, NaOH can be added during operation. An advantageous option is to use sodium sulfate (Na_2_SO_4_) as the secondary feed, since the Na^+^ ions do not affect the pH when transferred to the chelate recovery chamber. Addition of NaOH during operation only controls the bulk pH, while the use of Na_2_SO_4_ ensures a more stable pH in the diffusive layer. Na_2_SO_4_ is also a readily available by-product from today’s recycling processes [32].

Dechelation and product recovery is another challenge in this type of process. EDTA has previously been removed by acidic precipitation, enabling internal recycling back to the chelation stage. However, this method is not optimal since total dechelation is not achieved [23,29]. Fenton processes [33] and a method with thermally activated persulfate (S_2_O_8_^2−^) for oxidation of EDTA chelates are alternative methods. Carbon dioxide (CO_2_), water (H_2_O), ammonium (NH_4_^+^), and nitrate (NO_3_^−^) were proposed as final breakdown products of EDTA after reaction with persulfate ions [34].

Previous studies have focused on valuables and the behavior of leachate impurities has not been reported. Impurities are of paramount importance, as they are a potential cause of membrane fouling, contaminate products, and could be harmful to the environment. A holistic view of the process that considers solution purification, metal separation, and recovery is therefore needed. This study seeks to demonstrate better refining by electrodialysis through separation at higher metal concentrations than previously reported using a process based on Na_2_SO_4_ instead of H_2_SO_4_. It also investigates metal recovery after separation by using the method of thermally activated persulfate instead of acidic precipitation for dechelation. Leachate impurities, their behavior during electrodialysis, and their effect on product quality are also considered.

## 2. Materials and Methods

### 2.1. Chemicals

The chemicals used in this study were as follows: >99% sodium sulfate decahydrate (Na_2_SO_4_∙10H_2_O), >98% nickel(II) sulfate hexahydrate (NiSO_4_∙6H_2_O), >98% cobalt(II) sulfate heptahydrate (CoSO_4_∙7H_2_O), >98% manganese(II) sulfate monohydrate (MnSO_4_∙H_2_O), >99% lithium sulfate monohydrate (Li_2_SO_4_∙H_2_O), >99% ethylenediaminetetraacetic acid (EDTA), >97% sodium hydroxide (NaOH), >98% sodium sulfide nonahydrate (Na_2_S∙9H_2_O), and >98% sodium persulfate (Na_2_S_2_O_8_) from Thermo Scientific Chemicals (Stockholm, Sweden); and >96% sulfuric acid (H_2_SO_4_), 30% (*w*/*v*) hydrogen peroxide (H_2_O_2_), and >85% phosphoric acid (H_3_PO_4_) from VWR Chemicals (Kista, Sweden).

### 2.2. Electrodialysis Setup

An ED64004 unit from PCCell GmbH (Marpingen, Germany) was used for the electrodialysis tests. It has an active membrane area of 64 cm^2^ with a Pt/Ir-MMO coated Ti anode and a stainless steel (V4A) cathode. The membrane stack consisted of PC MTE CEM (end membranes), PC SK CEM, and PC Acid 60 AEM, all from PCCell GmbH. The end membranes were selected since they tolerate the pressure difference at the stack ends, as noted by the manufacturer.

The power source was a PGSTAT302N potentiostat from Metrohm Autolab BV (Bromma, Sweden). Four Masterflex L/S analog variable-speed console drives with Easy-Load II pump heads were used for pumping. Conductivities and pH were measured by three Orion Star A325 meters from Thermo Fisher Scientific Inc. (Stockholm, Sweden).

### 2.3. Equilibrium Calculations

Three different concentrations were studied to find the proper conditions for Ni separation by electrodialysis: EDTA (H_4_Y) additions equal to 0.5, 1.1, and 1.2 times the molar amount of Ni in the solution ($n_{EDTA}/n_{{Ni}^{2+}}$). The scenarios were modelled in HSC Chemistry 10.4.2.2. The feed was a solution of 0.10 M NiSO_4_, 0.10 M CoSO_4_, 0.10 M MnSO_4_, and 0.075 M Li_2_SO_4_ with a pH varying from 0 to 6. A Pitzer equation based on the Aqua model in HSC was used to calculate the activity coefficients. Data were taken from the HSC Chemistry 10.4.2.2. database, except for the metal-EDTA species, whose thermodynamic properties were retrieved from the National Institute of Standards and Technology (NIST) database [35]. Chelates with Li^+^ and EDTA were neglected due to their low stability (lg K = 2.95 for Li^+^ versus lg K = 18.4, 16.45, and 13.89 for Ni^2+^, Co^2+^, and Mn^2+^, respectively). The assumption is supported by the findings of Chan et al. [25], showing negligible chelation with Li^+^ at an acidic pH. The metal sulfate complexes (lg K = 2.4, 2.4, and 2.3 for Ni^2+^, Co^2+^, and Mn^2+^, respectively) formed were omitted from the model, assuming they gradually transform into metal cations migrating from the solution during operation. Sulfate chelation does not interfere with EDTA chelates, having profoundly higher stability constants (for example, reaction $NiY^{2-}\left(aq\right)+{SO}_{4}^{2-}\left(aq\right)⇄NiSO_{4}\left(aq\right)+Y^{4-}(aq)$ having a lg K value of −16).

**Figure 2:**
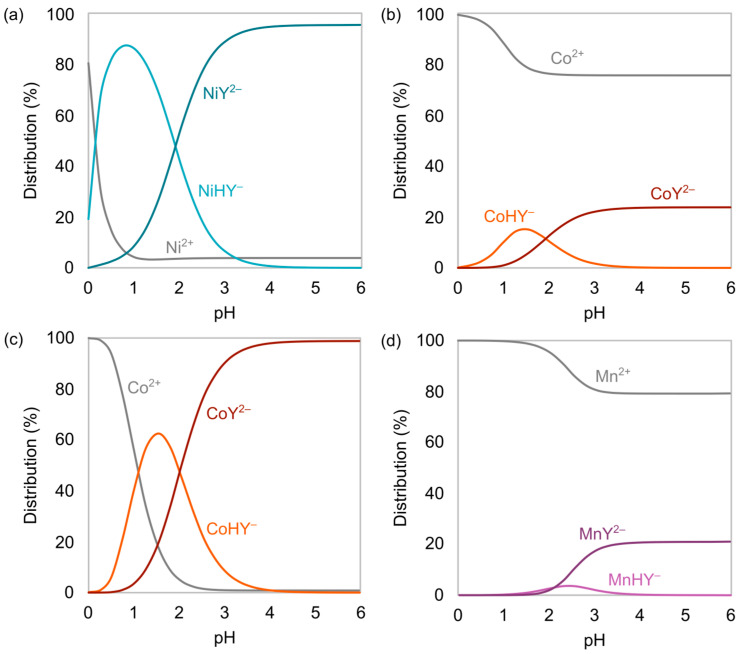
Speciation of Ni (**a**) and Co (**b**) with an EDTA addition equal to 1.2 times the Ni molar amount, as well as Co (**c**) and Mn (**d**) speciation upon Ni removal and an EDTA addition equal to 1.1 times the Co molar amount.

The results for the 1.2 scenario can be seen in Figure 2. Chelation of Mn^2+^ was negligible over the entire pH scale. The unchelated Ni^2+^ reached its minimum concentration at pH 1. At higher pH, NiHY^−^ was transformed into NiY^2−^. The amount of unchelated Ni^2+^ was almost constant at 3.9% from pH 1 to 6, implying 96.1% of Ni forms negatively charged species recoverable by electrodialysis. Most of the Co was present as Co^2+^ due to its slightly less stable EDTA chelates [23]. All Co existed as unchelated Co^2+^ at pH 0, and at pH 2, about 23.5% was bound to EDTA as CoHY^−^ and CoY^2−^. The speciation of Co and Mn without Ni is also shown in Figure 2. At pH 3.5, the share of unchelated Co^2+^ reached its minimum of 0.8%, indicating 99.2% of Co is recoverable by electrodialysis.

### 2.4. Separation of a Synthetic Leachate

Electrodialysis of a synthetic leachate was performed to verify the simulation results and to define test conditions with a black mass leachate. A four-chamber setup, as seen in Figure 1, with five repeating units was used for these tests.

A 0.33 M Na_3_HY solution was prepared by dissolution of solid EDTA in 1 M NaOH and mixed with the synthetic leachate for a solution corresponding to the HSC simulations. The pH was adjusted to 2.0 by adding 1 M H_2_SO_4_ before heating the mixture to 80 °C for 10 min, followed by cooling to 20 °C (room temperature) overnight, ensuring equilibrium. A low pH gives more selective chelation with Ni^2+^, but a pH lower than 2.0 is difficult due to small-sized H^+^ ions being readily transferred across the membranes [10], meaning they compete with metal ions for membrane transport.

**Figure 3:**
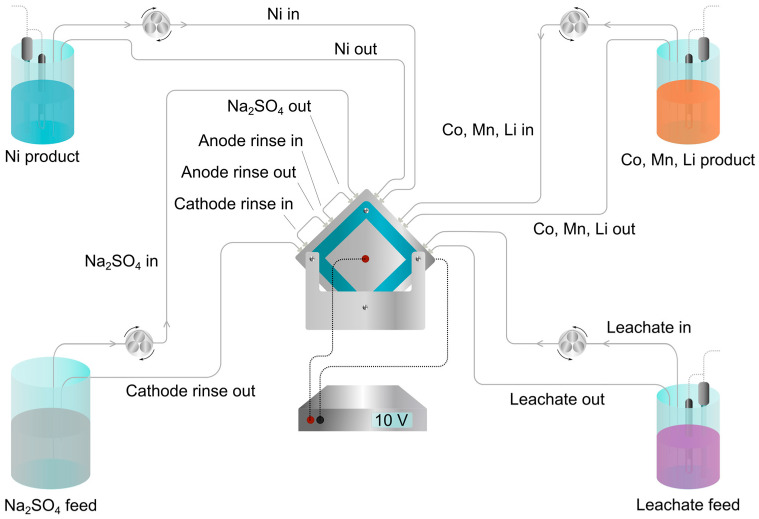
The experimental setup during electrodialysis. Ion exchange occurs between the leachate and a Na_2_SO_4_ solution to produce a Ni product and a Co, Mn, Li product. A Co product could then be produced by introducing the Co, Mn, Li product after Co chelation at the initial leachate position.

250 cm^3^ chelated synthetic leachate and 500 cm^3^ 0.50 M Na_2_SO_4_ were used as feed solutions. Ions migrate across the membranes, forming Ni-rich and Co, Mn, and Li-rich products, as shown in Figure 1. The same Na_2_SO_4_ solution was used as a rinse solution for the electrodes, which can be seen in the process overview in Figure 3. The product start solutions were 200 cm^3^ of 0.10 M H_2_SO_4_. This procedure was replicated with 0.50 M H_2_SO_4_ instead of 0.50 M Na_2_SO_4_ to compare pH and the extent of EDTA precipitation.

The stack voltage was 10 V with an initial current density of 200 A/m^2^. All solutions were pumped at 20 dm^3^/h, and flow rates were verified before electrodialysis by the time taken to fill a 100 cm^3^ graduated cylinder. Separation was considered complete when the leachate conductivity had dropped from approximately 40 to 0.3 mS/cm. Solutions were collected by rinsing the system with ultrapure water to a total product volume of 500 cm^3^, and samples were analyzed by inductively coupled plasma optical emission spectroscopy (ICP-OES).

Similar tests were conducted with a feed solution of 0.10 M CoSO_4_, 0.10 M MnSO_4_, and 0.075 M Li_2_SO_4_ to find proper EDTA addition for Co separation. The starting pH was in this case 3.5 rather than 2.0 due to the lower stability of the Co-EDTA chelates, as seen in Figure 2.

### 2.5. Preparation of an NMC 111 Leachate

A sample of NMC 111 (equimolar composition of Ni, Mn, and Co or LiNi_0.33_Mn_0.33_Co_0.33_O_2_) black mass provided by Stena Recycling AB (Gothenburg, Sweden) was sized at 420 µm by manual dry sieving to remove Cu and Al flakes ending up in the material upon mechanical LIB recycling. Fines (50.0 g) were mixed with 500 cm^3^ 1.0 M H_2_SO_4_ and 83 cm^3^ 30% (*w*/*v*) H_2_O_2_. The mixture was agitated at 80 °C for one hour before filtering.

Impurities were removed from the leachate in a two-stage procedure. The first stage was precipitation of Cu^2+^ by adding 0.20 M Na_2_S at pH 1.8 and 20 °C, added dropwise to prevent formation of hydrogen sulfide (H_2_S) gas. Samples were repeatedly taken from the leachate and analyzed with X-ray fluorescence (XRF) to follow the Cu content in the solution. Addition was stopped and the mixture filtered when the Cu content equaled 40 mg/dm^3^.

The remaining impurities, primarily Al, P, and Fe, were removed by hydroxide precipitation. NaOH solution (1 M) was added dropwise to the leachate until pH 4.5, followed by filtration. The Cu and Al, P, and Fe precipitates were dried overnight at 50 °C, digested in aqua regia, and analyzed together with the purified solution by ICP-OES.

### 2.6. Separation of Ni from the NMC 111 Leachate

Na_3_HY solution (0.33 M) was added to a 250 cm^3^ sample of purified leachate. The solution was added in a 20% excess of EDTA to the Ni molar amount, corresponding to the 1.2 scenario. The pH was adjusted to 2.0 by adding 1 M H_2_SO_4_, then heated, cooled, and separated by electrodialysis in the same way as the synthetic leachate. The procedure was replicated once.

As shown in the HSC simulations and reported in the literature [21,23,25], some Co is co-recovered when Ni recovery is maximized. This Co was removed by adding an equimolar amount of NiSO_4_∙6H_2_O, dechelating Co^2+^ and making it available for removal by electrodialysis. The solution was separated as in the first Ni recovery step.

### 2.7. Separation of Co from the NMC 111 Leachate

Two 250 cm^3^ batches of the Co, Mn, and Li products were mixed with 0.33 M Na_3_HY to form Co-EDTA chelates. Na_3_HY solution was added in a 10% excess of EDTA to the Co molar amount, corresponding to the 1.1 scenario. The mixture was heated to 80 °C for 10 min and then cooled to 20 °C, ensuring equilibrium before separation. The pH was adjusted to 3.5 before heating. Co was then separated in the same way as previously described for the synthetic leachate.

### 2.8. Energy Requirements

The specific energy demand E and current efficiency η for separation were calculated using Equations (1) and (2). Here, U is stack voltage, t operating time, I electric current, $n_{i}$ product molar amount of the target ion, z target ion absolute charge (assuming z=1.5, as pH 2.0 results in a mixture of monovalent and divalent chelates, see Figure 2), F the Faraday constant (96,485 As/mol), and N the number of repeating units.

(1) $$E=\frac{U\int_{0}^{t}Idt}{n_{i}}$$

(2) $$\eta =\frac{n_{i}zF}{N\int_{0}^{t}Idt}$$

### 2.9. Dechelation and Metal Recovery

The method with thermally activated persulfate was used to liberate Ni^2+^ and Co^2+^ from EDTA. Sodium persulfate (Na_2_S_2_O_8_) was added to the solutions in a metal:persulfate molar ratio of 1:30. The pH was adjusted to 1.0 with 1 M H_2_SO_4_. The mixture was heated to 80 °C for one hour to promote oxidation of EDTA.

Ni^2+^ and Co^2+^ ions were precipitated after dechelation by adding 5 M NaOH until pH 11.0. Mn^2+^ was recovered from the solution after Co recovery by adding 1 M NaOH solution until pH 11.0. A 10 cm^3^ sample of 30% H_2_O_2_ was added before NaOH to promote precipitation of MnO_2_ instead of Mn(OH)_2_, as the former ought to be easier to filter. Phosphate precipitation was used for Li recovery by adding 20 cm^3^ of 85% H_3_PO_4_ to the filtrate upon Mn recovery, and the pH was then adjusted to 12.5 by adding 5 M NaOH. The solution was heated to 50 °C and agitated for three hours to promote lithium phosphate (Li_3_PO_4_) precipitation and allowed to cool down to 20 °C overnight before filtration. All precipitates were analyzed by X-ray powder diffraction (XRD) and later digested in aqua regia for analysis by ICP-OES.

### 2.10. Sample Analysis

All samples collected for ICP-OES analysis were diluted 100 or 1000 times with 0.30 M HNO_3_ and analyzed on an iCAP 7000 Series ICP-OES unit from Thermo Fischer (Stockholm, Sweden). The XRF analyses were performed on a Spectro Xepos XRF. A Panalytical Empyrean (Uppsala, Sweden) was used for X-ray powder diffraction (XRD) with the following settings: Cu Kα radiation, 45 kV accelerating voltage, 40 mA electron emission current, 2θ measurement range of 5–90°, step size of 0.0260°, and 30-min scanning time.

The weight fractions $w_{imp}$ of black mass impurities (Cu, Al, Fe, and P) ending up in the final products were calculated using Equation (3), where $m_{i}$ denotes mass of an impurity element (i= Cu, Al, Fe, or P) and m is the total product mass. It is further assumed that small contents of Ni, Co, Mn, or Li in the products are not considered as impurities, as these metals are to some extent mixed during downstream battery production.

(3) $$w_{imp}=\frac{m_{Cu}+m_{Al}+m_{Fe}+m_{P}}{m}$$

## 3. Results

### 3.1. Ni Separation and Recovery

Results for Ni separation from the synthetic leachate are shown in Table 1. They generally agree with the HSC findings, which suggested 96.1% recovery of Ni with a 23.5% recovery of Co for the 1.2 scenario. Recovery over time during Ni separation is found in the Supplementary Material, Figure S1.

The 1.1 scenario gave similar recovery of Ni with significantly lower Co recovery than the 1.2 scenario. The 0.5 scenario shows that some Co, Mn, and Li are recovered even at small EDTA additions. The results are comparable with the results for the NMC 111 leachate shown in Table 2. It was possible to replicate this test and obtain recoveries within ±1% from the first results. About 3.6 kWh/mol Ni was needed for separation with a current efficiency of 2.3%. The current evolution over time is visible in Figure S2 in the Supplementary Material.

It was possible to remove 75.8% of Co, 89.2% of Mn, and 68.6% of Li from the Ni product in the purification step; 99.7% of Ni was recovered after dechelation and precipitation, giving a final product of 38.5 wt% Ni. The precipitate was amorphous, meaning phases could not be identified with XRD.

### 3.2. Co Separation and Recovery

Results for the synthetic leachate are displayed in Table 3. There was an almost negligible difference in recovery for the 1.1 and 1.2 scenarios. A white precipitate was observed in the Co product, not observed in the aforementioned Ni product.

The corresponding results for the NMC 111 leachate are shown in Table 4. The recovery was lower compared with Co separation from the synthetic leachate. Results were replicable and gave recoveries within ±1% from the first results.

The Co precipitate contained 43.1 wt.% Co, and Co recovery in the dechelation and precipitation stage was 87.0%. The product was, just like the Ni product, amorphous, meaning no phases could be identified. The separation energy was 2.5 kWh/mol Co with a current efficiency of 3.3%. The current evolution over time is visible in Figure S3 in the Supplementary Material.

### 3.3. Mn, Li Separation and Recovery

Above 99.9% of Mn in the Mn, Li product was recovered to the Mn precipitate, having a Mn grade of 40.9 wt.% and being amorphous. The Li precipitate contained 16.0 wt.% Li, and its stage recovery equaled 83.8%. Its crystal structure corresponded to lithium phosphate (Li_3_PO_4_), while no other crystalline phases were observed. Table 5 shows a summary of all final products and product grades as found in the study.

### 3.4. Na_2_SO_4_ as Replacement for H_2_SO_4_

Separation was possible at 0.10 M of Ni, Co, and Mn for the synthetic leachate and 0.05 M for the NMC 111 leachate. The concentrations are up to 30 times higher than reported in the literature for NMC leachates using similar methods [25].

**Figure 4:**
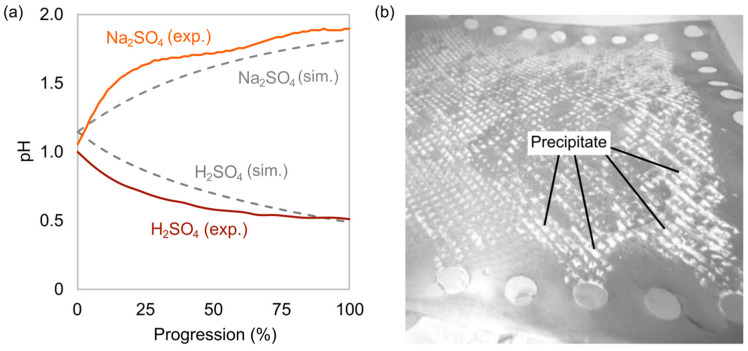
(**a**) Experimental and simulated pH of the Ni product using H_2_SO_4_ or Na_2_SO_4_ as secondary feed, showing electrodialysis progress (percent Ni recovered) for the 1.2 scenario with a synthetic leachate, (**b**) White precipitate on an AEM when H_2_SO_4_ was used as secondary feed.

Figure 4a shows the pH of the Ni product for the 1.2 scenario with the synthetic leachate together with the expected pH from HSC calculations. The initial pH was close to 1.0 in all cases due to the 0.10 M H_2_SO_4_ starting electrolyte. Figure 4b shows precipitation on an AEM when pH reached 0.5. The precipitate dissolved at pH 8.0 upon NaOH addition. When Na_2_SO_4_ was used instead of H_2_SO_4_, there was a pH increase in the solution, as electrodialysis progressed and no precipitation was observed.

### 3.5. Impurity Behavior

A mass balance for different elements present in the solutions is shown in Table 6. Cu removal by sulfide precipitation was near total (95.5%), while Cu remaining in the solution mainly ended up in the Ni product. Fe and P showed similar behavior, as much was removed by hydroxide precipitation (61.1% and 44.6%) while some followed Ni during electrodialysis. In general, sulfide precipitation was more effective at removing Cu than hydroxide precipitation was able to remove Fe, Al, and P. Most of the impurities ended up in the Ni product, but Al had a different behavior. It was more evenly distributed over the products, mostly ending up in the Co product. Purifying the Ni product in a second electrodialysis step had a small effect on impurity elements, and most of them followed Ni to the final Ni product.

## 4. Discussion

### 4.1. Electrodialysis for Separation of Ni and Co

The results show that electrodialysis is a technique suitable for separation of Ni and Co from black mass leachates, at least for equimolar composition black mass (NMC 111). The separation of metals was not complete, but they could be further refined by additional electrodialysis stages or selective precipitation. However, total separation of Ni, Co, and Mn may not be necessary, since these metals are mixed in different ratios during production of new NMC LIBs.

The separation recovery was higher for Ni than for Co due to the more stable Ni-EDTA chelates. The recoveries for the synthetic and NMC 111 leachates were more similar for Ni than for Co. Since the Co-EDTA chelates are less stable, any Ni remaining in the solution after Ni separation will compete with Co in binding to EDTA. Impurity elements such as Cu and Fe will compete in a similar way, meaning less Co is chelated and available for recovery by electrodialysis. A 10% molar excess of EDTA was used for Co chelation, but more EDTA could be needed to achieve higher recovery.

This study also shows that Na_2_SO_4_ is a viable alternative to H_2_SO_4_ as the secondary feed in a metathesis procedure. During electrodialysis, anions migrate from the leachate to combine with cations migrating from the secondary feed. When H_2_SO_4_ is used, pH decreases due to transfer of H^+^ ions, as evident from simulations and the experimental results. This means EDTA is protonated and dechelation occurs, leading to precipitation of EDTA. The white precipitate observed on the AEM in this study was not characterized but is most likely EDTA, further indicated by dissolution in weakly alkaline media. Metal precipitates would most likely not dissolve under these conditions. With Na_2_SO_4_, pH increased due to the buffering effect of SO_4_^2−^ ions and the chelate ions (reacting with H^+^ ions in solution forming their conjugate acids). Although Na_2_SO_4_ seems like an advantageous alternative, future studies should investigate more operating parameters, like the effect on current density and efficiency.

The precipitate in the Co product during Co separation could also be constituted of EDTA, since Co-EDTA chelates are less stable in acidic media, as seen in Figure 2. This is likely explained by the 0.10 M H_2_SO_4_ being used as a starting electrolyte, which could be too acidic for Co separation. A suggestion is to lower the concentration or use a different starting electrolyte, such as Na_2_SO_4_.

Although refining by electrodialysis works, the process is not energy efficient and showed a low current efficiency. A different AEM with larger pore size could aid the transport of metal-EDTA chelates, giving lower resistance and higher energy efficiency. A previous study on REE separation [28] has shown that large chelates could be sterically hindered from permeating the membranes. The current efficiency shows how much of the current is transported by Ni and Co chelates, but SO_4_^2−^ ions compete for current transport, lowering the efficiency. These ions cannot be totally removed from the process, but separation order could affect SO_4_^2−^ concentration and thus current efficiency. It would for example be advantageous to remove Li^+^ and Mn^2+^ as Li_2_SO_4_ and MnSO_4_ prior to Ni separation as these ions need to be transported twice across the CEM (during Ni and Co separation), lowering current efficiency. The influence of voltage and flow rates should also be further investigated to improve the energy efficiency of the process.

This study concerned NMC 111 black mass, but other types of NMC black mass are also of interest. Separation at a higher leachate concentration should be viable, especially for high-nickel black mass, which contains fewer unchelated divalent ions that are prone to precipitate during electrodialysis. Precipitation of EDTA is prevented using Na_2_SO_4_ as the secondary feed, which also enables separation at a higher concentration since more metal-EDTA chelates could be transferred to the product chambers. A potential problem is the EDTA excess needed for efficient Ni separation, since this excess binds to Co^2+^, forming Co-EDTA chelates that are co-recovered. A high Ni/Co ratio in the leachate may lead to poor separation of these metals. Further investigation with higher concentrations and other types of black mass is recommended.

### 4.2. Importance of Solution Purification

Cu, Fe, and P all followed Ni during electrodialysis. Since Ni is separated based on the negative charge of the Ni-EDTA chelates, it means impurities also formed negatively charged species. Cu^2+^ and Fe^3+^ both form stable EDTA chelates (lg K = 18.78 and 25.10, versus 18.40 for Ni^2+^) [35] and compete with Ni^2+^ for chelation. The presence of such chelates also explains why these impurities accompany Ni in two stages of separation. P is most likely present as PF_6_^−^ ions or phosphate species from PF_6_^−^ hydrolysis (PF_6_^−^ originating from the LIB electrolyte), all being negatively charged. Removal of Cu, Fe, and P is essential to achieve a pure Ni product. Sulfide precipitation effectively removed Cu from the leachate, but more Na_2_S must be added to further reduce the Cu content. More Co and Ni would likely be co-precipitated, but a mixed Cu, Ni and Co sulfide is a possible raw material for other refining processes. The hydroxide precipitate is more problematic since it likely has a lower economic value. Precipitation at a higher pH than 4.5 is desirable to remove more of the impurities, risking precipitation of Ni, Co and Mn.

Al had a different behavior in electrodialysis. It is found in most products, suggesting it forms differently charged species, such as negatively charged and uncharged EDTA chelates or hydroxo complexes. Since fluorine (F) exists in the black mass, there could possibly be different fluoro complexes, all generally being highly stable and of different charges [35]. F-containing species should be investigated in the future as they may affect product quality but also pose an environmental hazard. Al speciation is likely a combination of all these species, explaining the experimental results. Since Al was difficult to precipitate and ended up in all products, it should ideally be removed by mechanical methods before leaching.

### 4.3. Chelating Agents and Metal Recovery

Although it was possible to recover almost all Ni and Co bound to EDTA with the thermally activated persulfate method, it is not ideal from an economic-environmental perspective. Acidic precipitation is an inherently better method since it enables recirculation of EDTA, even though dechelation is not complete. One alternative could be to recover EDTA using acidic precipitation, followed by the method with thermally activated persulfate to remove residual metal-EDTA chelates. EDTA breakdown products such as NH_4_^+^ and NO_3_^−^ are problematic if extensive water treatment is required. Product grades were low, potentially because they constitute hydrates, double salts, or organics remaining from the dechelation process. More investigation is needed to fully understand which compounds were precipitated.

Another aspect that should be considered in the future are the kinetics of chelate formation, which are poorly understood but affect mixing time prior to electrodialysis. A slow chelation process may result in long mixing times, making the process upscale more difficult. The cooling-mixture procedure prior to electrodialysis may not be needed to ensure separation with high recovery rates. By omitting this step, the energy demand of the process can be lowered, making it more environmentally friendly and cost-effective.

Metal recovery after separation is the most challenging part of the process and should be investigated in future studies. The inability to recover and reuse EDTA makes the process more chemical-intense and adds to its overall environmental footprint.

## 5. Conclusions

In this study, it was shown that Na2SO4 can be used in an electrodialysis metathesis procedure to separate EDTA-chelated Ni and Co from black mass leachates. This was performed at 0.10 M (synthetic NMC 111 leachate) and 0.05 M (real NMC 111 leachate) of Ni, Co, and Mn respectively, up to 30-times higher concentrations than previously reported in the literature for this type of separation process using a black mass leachate. It is advantageous to use Na2SO4 as the secondary feed instead of H2SO4, which has previously been used, since Na+ ions do not decrease pH in product solutions, preventing precipitation of EDTA and allowing for separation at higher concentrations.

The recovery of Ni for the synthetic and NMC 111 leachates were 97.9% and 97.7%, respectively, at a 20% molar excess of EDTA with respect to Ni. The corresponding Co recoveries were 95.7% and 90.1% at a 10% molar excess of EDTA to Co. Remaining valuables, Mn and Li, could later be recovered by selective precipitation.

The major impurities in the leachate were Cu, Fe, P, and Al. A total of 95.5% of Cu was removed by sulfide precipitation before electrodialysis. The remaining Cu mainly followed Ni during separation, suggesting it was bound to EDTA as a negatively charged chelate. Fe and P also followed Ni during electrodialysis. Fe^3+^ forms strong EDTA chelates, while P originates from negatively charged PF_6_^−^ ions. Total removal of Cu, Fe, and P is paramount since they contaminate the Ni product.

Al ended up in all products during electrodialysis, which means it formed differently charged complexes. It was difficult to remove Al before electrodialysis without co-precipitation of valuables. It should ideally be removed before leaching.

Dechelation of EDTA after metal separation is difficult. The method with thermally activated persulfate was successfully used in this study, especially for Ni (99.7% precipitated from its chelate, compared with 87.0% for Co), but improved methods must be developed. The main drawback is that persulfate decomposes EDTA, so it cannot be recycled to the metal chelation stage, making the process more expensive and chemical-intense.

## Figures and Tables

**Table 1:** Metal recoveries from a synthetic leachate to the Ni product at different EDTA additions.

$\frac{n_{EDTA}}{n_{{Ni}^{2+}}}$	Ni rec.%	Co rec.%	Mn rec.%	Li rec.%
0.5	47.6	2.0	1.2	1.8
1.1	95.7	11.6	2.4	2.9
1.2	97.9	21.0	3.1	3.9

**Table 2:** Distribution of metals between different products in the Ni separation from the NMC 111 leachate.

Product	Ni dist.%	Co dist.%	Mn dist.%	Li dist.%
Diluate	0.7	0.2	0.1	0.0
Ni	97.7	21.4	1.8	2.1
Co, Mn, Li	1.6	78.4	98.2	97.9

**Table 3:** Metal recoveries from a synthetic leachate to the Co product at different EDTA additions.

$\frac{n_{EDTA}}{n_{{Co}^{2+}}}$	Co rec.%	Mn rec.%	Li rec.%
0.5	49.3	1.7	1.8
1.1	95.7	8.1	1.7
1.2	96.6	8.8	1.2

**Table 4:** Distribution of metals between different products in the Co separation from the NMC 111 leachate.

Product	Co dist.%	Mn dist.%	Li dist.%
Diluate	2.0	0.2	<0.1
Co	90.1	5.6	2.6
Mn, Li	7.8	94.2	97.4

**Table 5:** Product assays (*bdl* = below detection limit) and total impurity content $w_{imp}$ calculated using Equation (3).

Product	Ni wt.%	Co wt.%	Mn wt.%	Li wt.%	$w_{imp}$ wt.%
Ni precipitate	38.5	2.1	1.7	*bdl*	0.94
Co precipitate	1.0	43.1	2.3	*bdl*	0.26
Mn precipitate	0.1	3.5	40.9	*bdl*	0.25
Li precipitate	*bdl*	0.1	0.1	16.0	0.0010

**Table 6:** Simplified percent distribution of leachate elements upon solution purification and electrodialysis. Unrecovered (diluate) fractions have been added to the Co, Mn, Li; Mn, Li, and Co reject products.

Procedure	Product	Ni dist.%	Co dist.%	Mn dist.%	Li dist.%	Cu dist.%	Al dist.%	P dist.%	Fe dist.%
Solution purification	Leachate	100	100	100	100	100	100	100	100
	Cu precipitate	2.8	6.0	0.4	0.2	95.5	0.4	0.2	0.4
	Al, Fe, P precipitate	0.1	0.1	0.2	0.01	0.5	17.2	44.6	61.1
	Electrodialysis feed	97.1	93.9	99.4	99.8	4.0	82.4	55.2	38.5
Ni separation	Ni product	94.8	20.1	1.8	2.1	3.7	13.4	50.8	33.6
	Co, Mn, Li product	2.3	73.8	97.6	97.7	0.3	69.0	4.4	5.0
Ni purification	Purified Ni product	86.9	4.8	0.2	0.6	3.3	3.4	43.9	31.0
	Co reject	7.9	15.3	1.6	1.4	0.4	10.1	6.9	2.5
Co separation	Co product	2.3	66.5	5.5	2.5	0.01	46.9	0.7	0.01
	Mn, Li product	0.01	7.3	92.1	95.2	0.3	22.0	3.7	5.0

## Data Availability

The original contributions presented in this study are included in the article/Supplementary Material. Further inquiries can be directed to the corresponding author.

## Supplementary Materials

The following supporting information can be downloaded at: https://www.mdpi.com/article/10.3390/membranes15040097/s1, Figure S1: Evolution of molar amounts over time for different elements in feed and product compartments. The figure shows data for the Ni separation with the 1.2 scenario using a synthetic NMC 111 leachate, Figure S2: Recorded current during electrodialysis for Ni separation (1.2 scenario) using the synthetic NMC 111 leachate, Figure S3: Recorded current during electrodialysis for Co separation (1.1 scenario) using the synthetic NMC 111 leachate.
