# Supplementary material for: Nickel and Cobalt Recovery from Spent Lithium-Ion Batteries via Electrodialysis Metathesis
Source: Membranes (Basel). 2025 Mar 25;15(4):97. doi: 10.3390/membranes15040097 (PMC12028818; doi:10.3390/membranes15040097)
Supplement: Supplementary file 1 [file membranes-15-00097-s001.zip › membranes-3485571-supplementary.pdf]

membranes

MDPI

Article

Nickel and Cobalt Recovery from Spent Lithium-Ion Batteries via Electrodialysis Metathesis

Adam Isaksson [1,*], Juan Anaya Garzon [2], Ida Strandkvist [1] and Lena Sundqvist Öqvist [1]

[1] Division of Minerals and Metallurgical Engineering, Luleå University of Technology, 971 87 Luleå, Sweden; ida.strandkvist@ltu.se (I.S.); lena.sundqvist-oqvist@ltu.se (L.S.Ö.)
[2] Northvolt Revolt AB, 721 36 Västerås, Sweden; juandavid.anaya-garzon@northvolt.com
* Correspondence: adam.isaksson@ltu.se

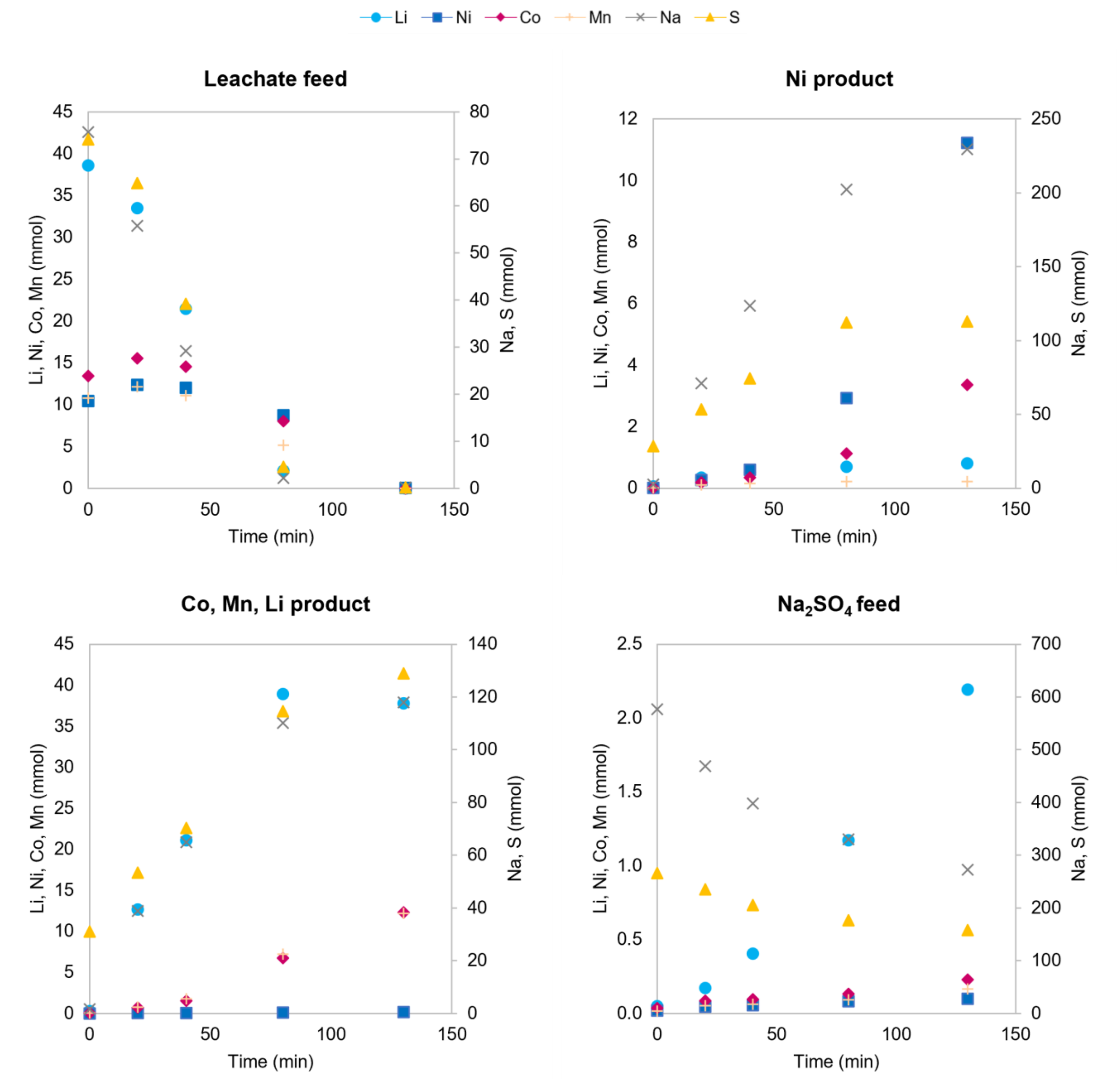

Figure S1. Evolution of molar amounts over time for different elements in feed and product compartments. The figure shows data for the Ni separation with the 1.2 scenario using a synthetic NMC 111 leachate.

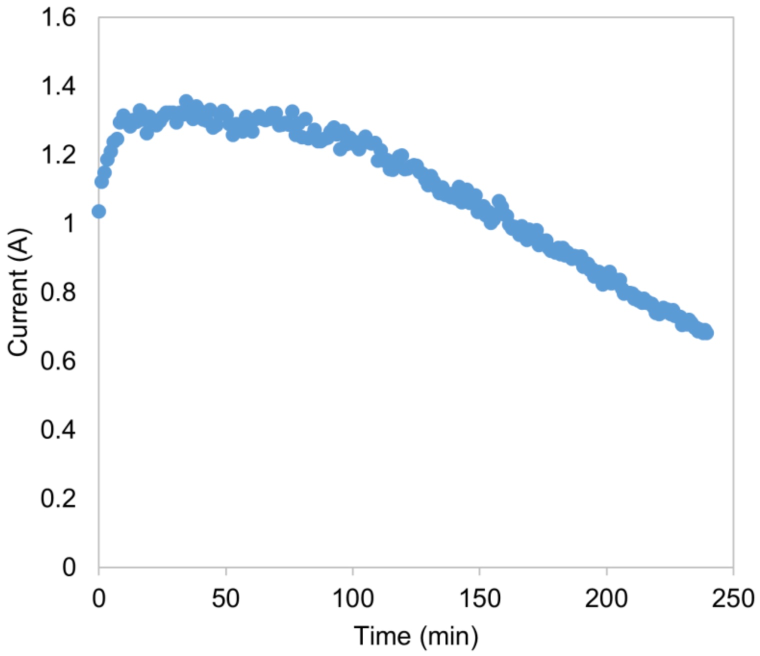

Figure S2. Recorded current during electrodialysis for Ni separation (1.2 scenario) using the synthetic NMC 111 leachate.

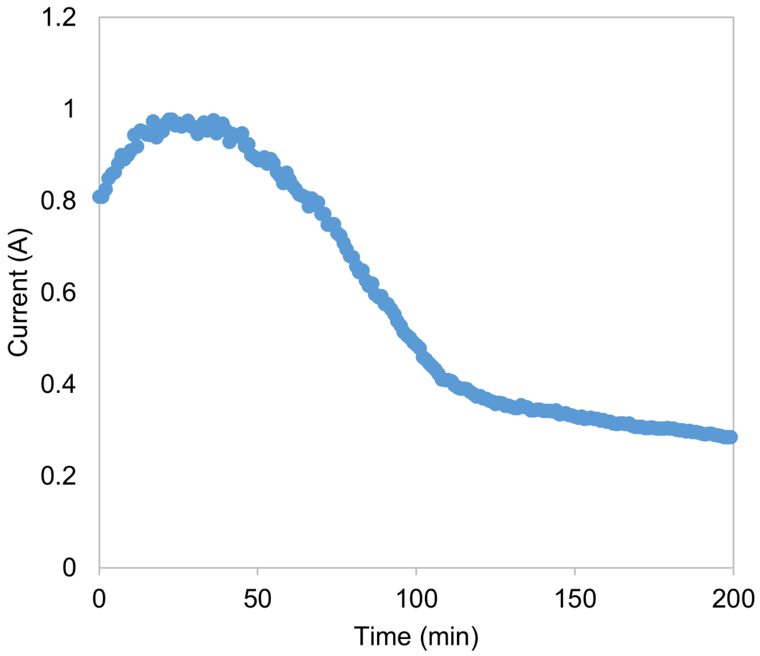

Figure S3. Recorded current during electrodialysis for Co separation (1.1 scenario) using the synthetic NMC 111 leachate.
